# Improvement of the Thermal Stability and Aqueous Solubility of Three Matrine Salts Assembled by the Similar Structure Salt Formers

**DOI:** 10.3390/ph17010094

**Published:** 2024-01-10

**Authors:** Yeyang Wang, Baoxi Zhang, Wenwen Wang, Penghui Yuan, Kun Hu, Li Zhang, Dezhi Yang, Yang Lu, Guanhua Du

**Affiliations:** 1 Beijing City Key Laboratory of Polymorphic Drugs, Center of Pharmaceutical Polymorphs, Institute of Materia Medica, Chinese Academy of Medical Sciences and Peking Union Medical College, Beijing 100050, China; 2 Shandong Province Key Laboratory of Polymorphic Drugs, Shandong Yikang Pharmaceutical Co., Ltd., Tengzhou 277500, China; 3 Beijing City Key Laboratory of Drug Target and Screening Research, National Center for Pharmaceutical Screening, Institute of Materia Medica, Chinese Academy of Medical Sciences and Peking Union Medical College, Beijing 100050, China

**Keywords:** matrine, salt, stability, solubility, theoretical calculation

## Abstract

Matrine (MAT), a natural Chinese herbal medicine, has a unique advantage in the treatment of various chronic diseases. However, its low melting point, low bioavailability, and high dosage restrict its subsequent development into new drugs. In this study, three kinds of MAT salts, namely, MAT-2,5-dihydroxybenzoic acid (MAT-25DHB), MAT-2,6-dihydroxybenzoic acid (MAT-26DHB), and MAT-salicylic acid-hydrate (MAT-SAL-H_2_O), were designed and synthesized to improve the drugability of MAT. The three salts were characterized by using various analytical techniques, including single-crystal X-ray diffractometry, powder X-ray diffractometry, differential scanning calorimetry, thermogravimetry, and infrared spectroscopy. The results of the thermal stability evaluation showed that the formation of salts improved the stability of MAT; MAT-25DHB is the most stable salt reported at present. The results of aqueous solubility showed that the solubility of MAT-25DHB was higher than that of MAT, while that of MAT-26DHB and MAT-SAL-H_2_O were less. Given that the MAT-25DHB salt further improved the solubility of MAT, it is expected to be subjected to further research as an optimized salt. Lattice energy and solvation free energy are important factors affecting the solubility of salts; the reasons for the changes of solubility and stability of three kinds of salts are explained by calculating them.

## 1. Introduction

**Figure 1:**
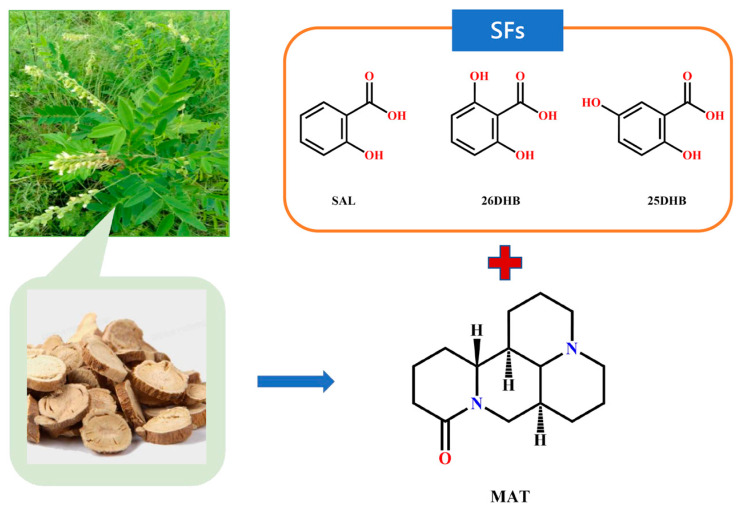
*S. flavescens* and the chemical structures of MAT and salt formers.

Matrine (MAT) is a tetracyclic quinoline alkaloid extracted and isolated from the roots and fruits of *Sophora flavescens* (Figure 1) [1]. It has a variety of pharmacological effects, such as antiinflammation effects, oxidative stress reduction, autophagy and apoptosis regulation, and cancer cell metastasis inhibition [2,3,4,5]. Numerous studies have shown that MAT has therapeutic effects on multiple sclerosis, Alzheimer’s syndrome, and other neurological disorders [6,7,8]. MAT is a member of the class I of the biopharmaceutics classification system and has the advantages of extensive sources, high safety, low price, and convenient administration [9,10,11]. However, due to its low melting point, poor activity, and high dosage, it is easy for it to induce adverse reactions such as liver toxicity and neurotoxicity, which limits its clinical application [12,13,14]. Therefore, improving the bioavailability of MAT is essential for its subsequent research and application.

Previously reported methods for improving the bioavailability of MAT include the modification of drug structures and the preparation of compounds, such as MAT derivatives and injectable *S. flavescens* compounds [15,16,17]. Cocrystal or salt formation is an effective method for improving the physicochemical properties and pharmacokinetic properties of drugs without changing their chemical structures [18,19,20,21,22]. The formation of salt is similar to the formation of cocrystals, which are complexes formed by the transfer of protons from acidic components to alkaline components and the combination of the active pharmaceutical ingredient (API) and cocrystal formers (CCFs) or salt formers (SFs) through hydrogen bonding [23,24,25]. Previous studies have demonstrated that MAT can form salts with acids by proton transfer reaction while maintaining its biological activity [26,27]. Mi et al. [26] formed MAT salts with syringic acid (SYR), vanillic acid (VAN), protocatechuic acid (PRO), caffeic acid (CAF), and gallic acid (GAL). The obtained salts showed improved anticancer, antioxidant, and anti-inflammatory properties. The biological activity of MAT greatly improved through the formation of a salt with salicylic acid (SAL) [28].

Benzoic acid compounds are recognized as safe (GRAS) compounds approved by the US Food and Drug Administration (FDA) and are often used as additives in food, cosmetics, pharmaceutical products, and hygiene products [29]. In this study, benzoic acid compounds were selected as the SFs to form salt with MAT. This method is expected to further improve the aqueous solubility of MAT, which can further improve its bioavailability and reduce its dosage while achieving the same therapeutic effect. If the adverse reactions of MAT are dose-dependent, the reduction of adverse reactions can be expected with the reduction of dosage. In addition, the benzoic acid compounds selected in this study have high melting points and have the potential to improve the thermal stability of MAT. We finally obtained three new salts, namely, MAT-2,5-dihydroxybenzoic acid (MAT-25DHB), MAT-2,6-dihydroxybenzoic acid (MAT-26DHB), and MAT-salicylic acid-hydrate (MAT-SAL-H_2_O). The salts were analyzed and characterized by various techniques. All three salts showed excellent thermal stability. The melting points of MAT-25DHB and MAT-26DHB were higher than those of the SFs and MAT, and they are the most stable salts reported so far. A crystal packing similarity analysis was conducted due to the similar crystallographic parameters of the three salts. In addition, the solubility of the three salts in four different pH buffer aqueous solutions was investigated. The results of this investigation provided some basis for the further study of MAT salts. Finally, theoretical calculations were performed to explain the changes in thermal stability and aqueous solubility from the perspective of energy, thus providing some references for the further study of MAT.

## 2. Results and Discussion

### 2.1. Crystal Structure Analysis

**Figure 2:**
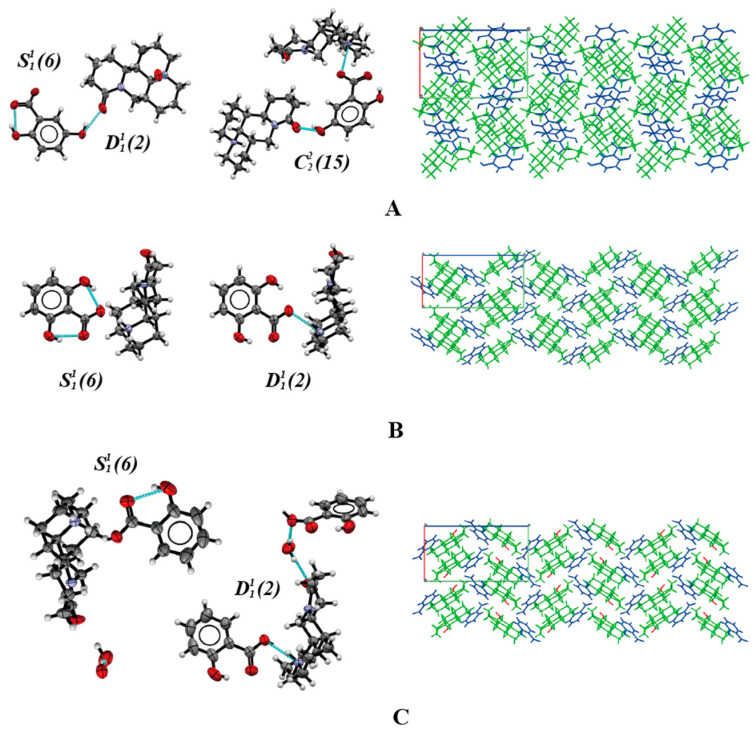
Hydrogen bond contacts (**left**) and packing of cocrystal structures (**right**; green: MAT, blue: SFs, red: H_2_O). (**A**) MAT-25DHB; (**B**) MAT-26DHB; (**C**) MAT-SAL-H_2_O.

Three kinds of salt crystals suitable for single-crystal X-ray diffraction (SCXRD) were prepared by at slow evaporation method in different solvents. The crystallographic data and structural refinement parameters of the three salts are shown in Table 1. The analysis of the structural characteristics of the three salt types shows that the three salts all belong to the orthorhombic space group *P* 2_1_2_1_2_1_ (Z = 4). The MAT-25DHB asymmetric unit contains one MAT and one 25DHB; the MAT-26DHB asymmetric unit contains one MAT and one 26DHB; and the MAT-SAL-H_2_O asymmetric unit contains one MAT, one SAL, and one water molecule. The three-dimensional structure diagram and the corresponding arrangement law of the three salts are shown in Figure 2. The main hydrogen bond parameters are listed in Table 2. The analysis shows that the nitrogen atoms in the MAT structure form hydrogen bonds with the oxygen atoms on the carboxyl groups of the three acids. In accordance with the position of the hydrogen atoms in the crystal [30] the hydrogen atoms on the carboxyl group in the three SF structures are transferred to the N_2A_ of the MAT molecule, indicating that proton transfer occurs between API and SF, and the three obtained solids are salts. The characteristics and differences of hydrogen bond modes in the three kinds of salt crystal structures were analyzed by graph set analysis. In MAT-25DHB, the N_2A_ in the hydrogen bond motif of the MAT molecule **$D_{1}^{1}$*(2)*** forms hydrogen bonds with the hydroxyl groups in the carboxyl groups of 25DHB, and the O_1A_ in the hydrogen bond motif of the same MAT molecule **$D_{1}^{1}$*(2)*** forms hydrogen bonds with the hydroxyl groups in position five of the different 25DHB molecules. In addition, the hydroxyl groups in the carboxyl groups of the 25DHB molecules and the hydroxyl groups in position two form intramolecular hydrogen bonds in the **$S_{1}^{1}$*(6)*** hydrogen bond motif. The hydroxyl group in the carboxyl group of the 25DHB molecule and the hydroxyl group in position two form hydrogen bonds with the N_2A_ and O_1A_ of the MAT molecule in the **$C_{2}^{2}$*(15)*** hydrogen bond motif. In MAT-26DHB, the N_2A_ in the hydrogen bond motif of the MAT molecule **$D_{1}^{1}$*(2)*** forms hydrogen bonds with the hydroxyl groups in the carboxyl groups of 26DHB. In addition, the hydroxyl group in the carboxyl group of the 26DHB molecule forms intramolecular hydrogen bonds with the hydroxyl groups in positions of two and six, respectively, in the **$S_{1}^{1}$*(6)*** hydrogen bond motif. In MAT-SAL-H_2_O, the O_1A_ in the hydrogen bond motif of the MAT molecule **$D_{1}^{1}$*(2)*** forms hydrogen bonds with the hydroxyl groups of the water molecules; the N_2A_ in the hydrogen bond motif of the MAT molecule **$D_{1}^{1}$*(2)*** forms hydrogen bonds with the hydroxyl groups in the carboxyl groups of salicylic acid molecules. The hydroxyl groups of the water molecules form hydrogen bonds with the hydroxyl groups in the carboxyl groups of different SAL molecules in the **$D_{1}^{1}$*(2)*** hydrogen bond motif. The hydroxyl group in the carboxyl group of the SAL molecule and the hydroxyl group in position two form intramolecular hydrogen bonds in the **$S_{1}^{1}$*(6)*** hydrogen bond motif. It is worth noting that the hydrogen bonding modes of MAT-25DHB and MAT-SAL-H_2_O are similar. Compared with the others, the number of intramolecular hydrogen bonds is highest in MAT-26DHB. The interaction between the API and SFs plays a key role in maintaining the whole hydrogen bonding network.

MAT-25DHB forms a network arrangement along the c-axis through the hydrogen bond connection of N_2A_–H_2A_…O_4B_ and O_1B_–H_1B_…O_1A_, and the intermolecular connection of MAT-26DHB forms a wavy arrangement along the c-axis through the hydrogen bond of N_2A_–H_2A_…O_2B_. MAT-SAL-H_2_O forms a wavy arrangement along the c-axis through the hydrogen bond connection of N_2A_–H_2A_…O_1B_ and O_1W_–H_1W_…O_1A_.

### 2.2. Hirshfield Surface Analysis

**Figure 3:**
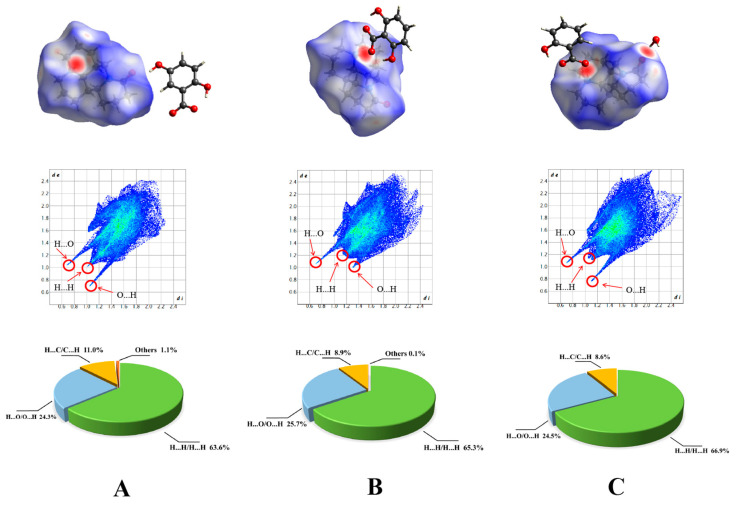
HS maps, 2D fingerprint plots, and percentage contributions to the HS area of three salts (**A**) MAT-25DHB; (**B**) MAT-26DHB; (**C**) MAT-SAL-H_2_O.

Hirshfeld surface analysis enables the visual analysis of molecular interactions in crystal structures [31,32]. The Hirshfeld surface analysis map and two-dimensional (2D) fingerprint plots are given in Figure 3. The red spot represents the hydrogen bond between the molecules, and the color shade directly reflects the strength of the intermolecular force. A dark color is indicative of a strong intermolecular force. Figure 3 shows that the hydrogen bond interaction sites of MAT-25DHB and MAT-SAL-H_2_O are concentrated at the O_1A_ and N_2A_ positions of MAT and the hydrogen bonding sites of MAT-26DHB are concentrated at the N_2A_ position of MAT.

The 2D fingerprint can reflect the type and proportion of the hydrogen bonds in crystals. A sharp peak is indicative of a strong action mode. The 2D fingerprints of the intermolecular forces of the three salts are shown in Figure 3. In the full spectrum, H…O/O…H and H…H/H…H contacts are the main components. H…O/O…H contacts account for 24.3%, 25.7%, and 24.5% of the intermolecular forces in MAT-25DHB, MAT-26DHB, and MAT-SAL-H_2_O, respectively, with the two sharp peaks located at de > di and di > de indicating that the O atom is present as a hydrogen bond donor and a hydrogen bond acceptor in the three structures. H…O/O…H has similar action forces in the three salts because the hydrogen bond is formed by the interaction between the N atom of MAT and the O atom of the three acids.

### 2.3. PXRD Analysis

**Figure 4:**
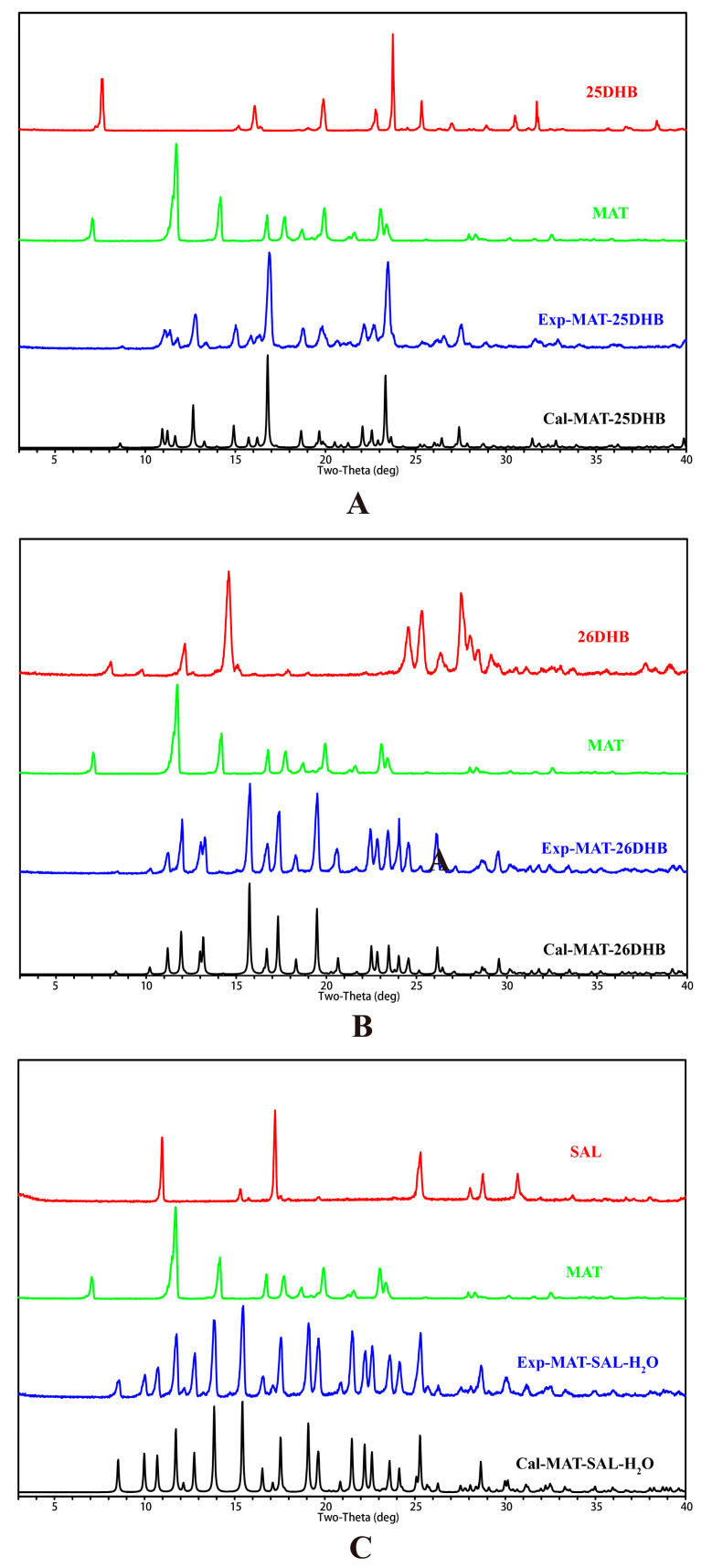
PXRD patterns of MAT, the SFs, and the corresponding salts. (**A**) MAT-25DHB; (**B**) MAT-26DHB; (**C**) MAT-SAL-H_2_O (black: calculated, blue: experimental, green: SFs, red: MAT).

Powder X-ray diffraction (PXRD) is an effective method for the qualitative identification and quantitative analysis of crystalline drugs in accordance with the number, intensity, and position of the diffraction peaks [33]. Figure 4 shows that MAT and the three acids have formed a new phase that is different from the API and SFs and that their diffraction peak position and peak intensity have changed. The main characteristic diffraction peaks of MAT and the three acids have disappeared, and new characteristic peaks have formed. The experimental PXRD patterns have a high degree of agreement with the calculated PXRD patterns, showing that the samples have high purity [34].

### 2.4. Crystal Packing Similarity Analysis

**Figure 5:**
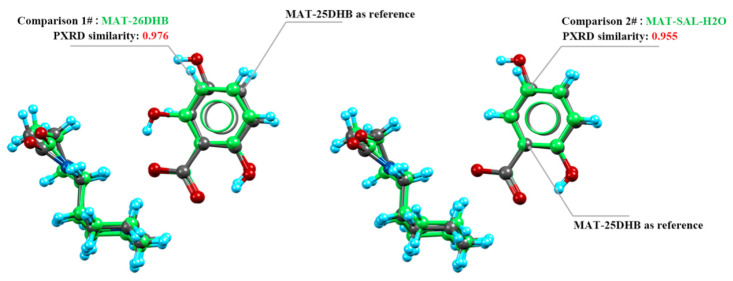
Crystal packing similarity of the MAT salts.

A crystal packing similarity analysis was conducted because the three salts have similar crystallographic parameters. The cumulative similarity between MAT-25DHB and the other two salts was calculated by using MAT-25DHB as the reference molecule (shown in green in Figure 5). The conformation of the MAT main molecule can intuitively be seen to be basically the same in the three salts, and the conformation of the guest molecule (benzoic acid part) is basically the same. The similarities of the calculation results obtained by using Mercury are as follows: the PXRD similarity between MAT-25DHB and MAT-26DHB is 0.976, and the similarity between MAT-25DHB and MAT-SAL-H_2_O is 0.955. Compared with the SFs, the influence of the hydroxyl number on the intermolecular force between API and SF is greater than that of the hydroxyl position, so that the similarity of MAT-26DHB is higher than that of MAT-SAL-H_2_O.

### 2.5. Thermal Analysis

**Figure 6:**
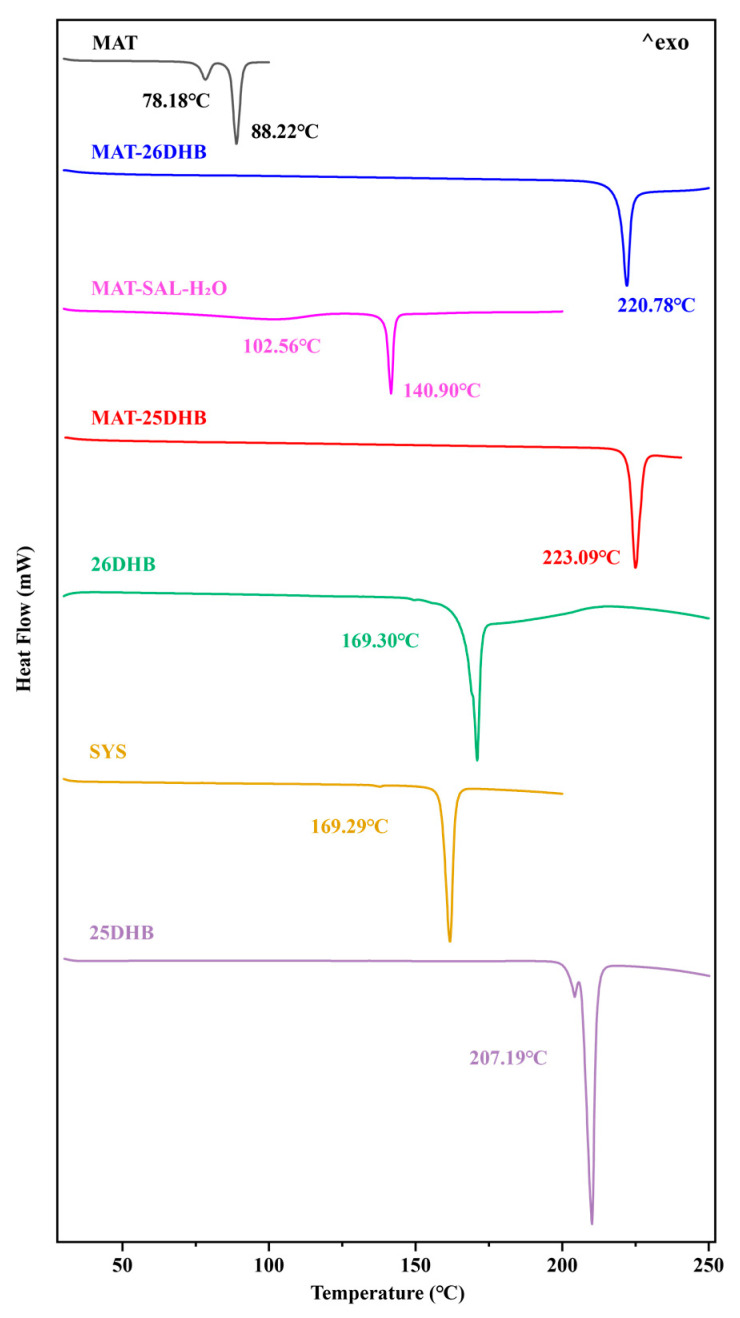
DSC curves of MAT, the SFs, and the corresponding salts.

Differential scanning calorimetry (DSC) is performed for the qualitative and quantitative analyses of different cocrystals with varying thermodynamic properties in accordance with the number, location, and enthalpy of the endothermic or exothermic peaks. The DSC analysis results show that the two MAT salts possess a single endothermic peak, and the MAT hydrate presents two sharp endothermic peaks. The peak temperatures of the three salts are different from those of MAT and the SFs, proving the formation of new substances. Figure 6 shows that the endothermic peak temperature of MAT-25DHB is 223.09 °C, which is higher than that of MAT (78.18 °C and 86.5 °C) and 25DHB (203.60 °C and 207.19 °C). The endothermic peak temperature of MAT-26DHB is 220.78 °C, which is also higher than that of MAT and 26DHB (169.30 °C). The endothermic peak temperatures of MAT-SAL-H_2_O are 102.90 °C and 140.90 °C, which are between those of MAT and SAL (160.29 °C).

**Figure 7:**
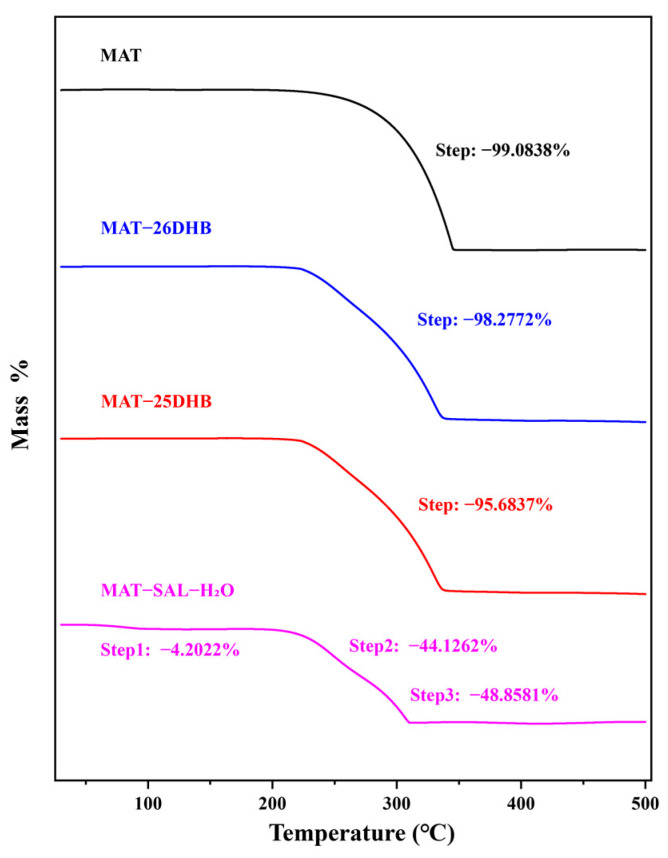
TG curves of MAT and the three salts.

A thermogravimetric analysis (TGA) was performed to determine the decomposition temperature of the salts and to identify the mass fraction of solvent in the samples on the basis of the proportion of mass lost by the substance to be measured in the corresponding range. The DSC and TG curves (Figure 7) illustrate that MAT-25DHB and MAT-26DHB do not contain solvent or water, MAT-SAL-H_2_O contains one molecule of water, and a weight loss step occurs at the corresponding melting point.

**Figure 8:**
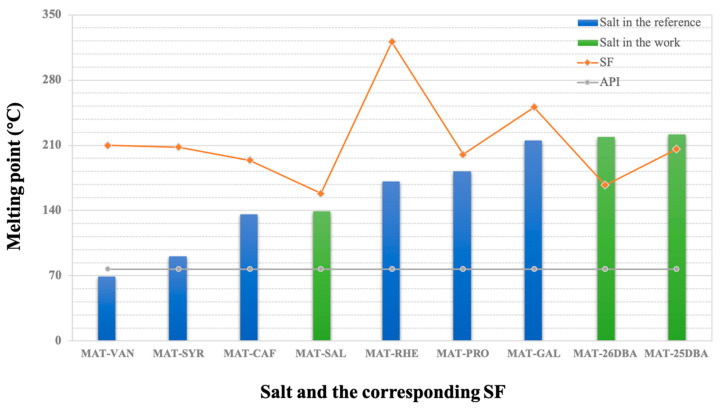
Melting point distribution diagram of the salts and the corresponding SFs.

Figure 8 presents the previously reported MAT salts. These salts are arranged from low to high on the basis of their melting points. No relationship is found between the melting point of the obtained salts and that of the SF. Generally, the high melting point means that the substance has good thermal stability [35]. The melting point of all salts, except MAT–VAL, is higher than that of MAT, suggesting that the formed salts have improved thermal stability. The melting points of MAT-25DHB and MAT-26DHB are higher than their corresponding SFs, and they are the most stable salts reported so far.

### 2.6. IR Analysis

**Figure 9:**
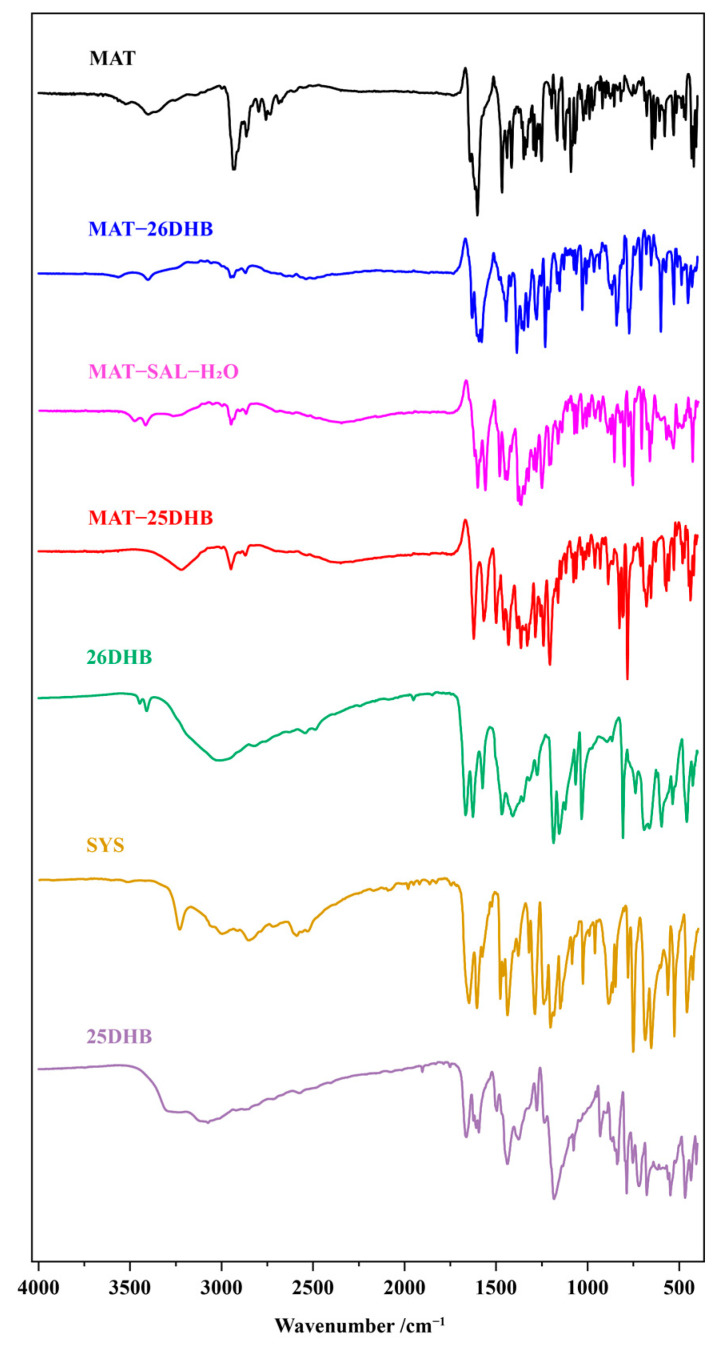
IR spectra of MAT, the SFs, and the corresponding salts.

Cocrystals or salts can be qualitatively analyzed through infrared spectroscopy (IR), and chemical bond absorption peaks shift when cocrystals or salts are formed. The carbonyl group, a characteristic functional group in infrared spectra, is often used to judge hydrogen bond formation. As shown in Figure 9, the carbonyl absorption peaks at 1662, 1667, and 1654 cm^−1^ in 25DHB, 26DHB, and SAL, respectively, shift to 1622, 1629, and 1604 cm^−1^ in MAT-25DHB, MAT-26DHB, and MAT-SAL-H_2_O, respectively, indicating that intermolecular interaction forces may change due to the formation of hydrogen bonds.

### 2.7. Equilibrium Solubility

Aqueous solubility is one of the key factors affecting the bioavailability of drugs [36]. The equilibrium solubility of MAT and its three salts in purified water, phosphate buffer (pH 6.8), acetic acid buffer (pH 4.5), and hydrochloric acid solution (pH 1.2) in 37 °C was determined by the shake flask method. Three parallel experiments were conducted, and the results are shown in Table 3. In pure water, the solubility of MAT-25DHB is higher with the hydrochloric acid solution (pH 1.2), acetate buffer (pH 4.5), and phosphate buffer (pH 6.8) than the solubility of MAT by 9.39, 8.75, 6.60, and 7.44 times, respectively; the solubility of MAT-26DHB is 0.11, 0.14, 0.09, and 0.09 times that of MAT, respectively; and the solubility of MAT-SAL-H_2_O is 0.44, 0.61, 0.44 and 0.39 times that of MAT, respectively. It can be seen that, compared with the solubility of MAT, the solubility of MAT-25DHB was significantly higher, whereas that of MAT-26DHB and MAT-SAL-H_2_O was lower. The results show that the formation of salt is not necessarily beneficial for improving the solubility of API. On the other hand, MAT is a weakly basic compound, and its degree of ionization in the solution is affected by the concentration of other ions in the solution [37,38]. Generally, the charged part has high aqueous solubility. Three salts have the highest solubility at pH 1.2, because the API and SFs are in an ionized state at that pH. We speculated that the reason for the solubility decrease of MAT in phosphate buffers is that the ions in the buffer inhibit the dissociation of MAT and thus reduce the solubility of the latter.

### 2.8. Theoretical Calculations

The solubility of cocrystals or salts can be regulated by two independent factors, namely, lattice energy and solvation free energy. First of all, molecules need to overcome the lattice energy and release from the lattice and then overcome the solvation free energy to complete the dissolution of solute molecules. Lattice energy can be reduced and/or solvation free energy can be increased in order to increase solubility [39]. Therefore, the change in solubility can be explained accordance to the corresponding calculations below.

#### 2.8.1. Lattice Energy

Lattice energy refers to the energy absorbed when a crystal transforms into gaseous positive and negative ions under standard conditions (0 K, 1 standard pressure). It can be used to measure the thermal stability of crystals [40]. Lattice energy can be calculated by using the following formula:

(1) $$∆E\left(lattice\right)=∆E\left(cond\right)+BSSE$$

where ∆E (lattice energy) is the lattice energy, ∆E (condensation) is the condensation of molecules that retain the same conformation they had in the crystal, and BSSE is the basis set superposition error.

(2) $$∆E\left(cond\right)=\frac{E\left(ulk\right)}{Z}-E\left(mol,bulk\right)$$

(3) $$BSSE=E\left(mol,bulk\right)-E\left(mole,ghost\right)$$

where E (bulk) represents the total energy per unit cell, and Z represents the number of molecules in the cell. E (molecule, bulk) represents the total energy of each molecule in the cell, and its geometry is consistent with that in the cell. E (molecule, ghost) represents the total energy of the molecule obtained by increasing the basis group with the virtual function of the surrounding atoms.

CRYSTAL 17 software was used to obtain the values of all items in the above formula, and the lattice energy of each salt was calculated in kcal/mol. The other terms were in Hartree. The results are shown in Table 4. The order of the lattice energy of the salts obtained in this work is consistent with the corresponding melting point order of the salts, that is, a large lattice energy (highly negative) corresponds to a high melting point. Compared with the MAT, the formation of salt makes MAT and the SF in the state of positive and negative ions, and the hydrogen bond formed between them increases in strength due to the assistance of electric charge, which increases the lattice energy. This correspondence explains the improvement in thermal stability from the perspective of energy.

#### 2.8.2. Solvation Free Energy

Solvation free energy ΔG (solv) is the free energy that transfers the solute from the gas phase to the solvent at constant temperature and pressure (the commonly used gas standard configuration is 1 atm and the temperature is 298.15 K.) Solvation free energy describes the difficulty of solute molecules dissolving in solvents and can be calculated by using the following equation:

$$∆G\left(solv\right)=E\left(soln\right)-E\left(gas\right)$$

where E (soln) represents the single-point energy in the solvent, and E (gas) represents the single–point energy in the gaseous state.

Calculation revealed that the solvation free energies of MAT-25DHB, MAT-26DHB, and MAT-SAL-H_2_O in water are −101.47, −27.00, and −30.32 kcal/mol. These results are in good agreement with the findings of the solubility experiment, which followed the order of MAT-25DHB > MAT-SAL-H_2_O > MAT-26DHB. The formation of salt makes the API and SF in the state of positive and negative ions, which makes them interact with the solvent more easily than the neutral molecules, thus improving the aqueous solubility. Moreover, they show that, although 25DHB and 26DHB are isomers, the solvation free energy of the former is significantly lower than that of the latter after salt formation with MAT. This result also explains the significant difference in solubility between the two salts. The variables appearing in the above formula are shown in the Table 5.

## 3. Materials and Methods

### 3.1. Materials

MAT raw material (98%) was purchased from Yanchi County Dushun Biology Chemical & Industrial Co. (Ningxia, China). The 2,5–Dihydroxybenzoic acid (25DHB, 98%), 2,6–dihydroxybenzoic acid (26DHB, 98%), and SAL (98%) were purchased from Hubei Wande Chemical Co. (Tianmen, China). All analytical-grade solvents were purchased from Fuchen Chemical Reagent Co. (Tianjin, China).

### 3.2. Methods

#### 3.2.1. Preparation

For the preparation of MAT-25DHB (1:1), 0.2 mmol MAT (49.6 mg) and 0.2 mmol 25DHB (30.8 mg) were weighed into a mortar and 1 mL of ethyl acetate was added for grinding to obtain a powder sample. The prepared powder sample was dissolved in a mixture of ethyl acetate and methanol (*v*/*v*, 4:1), filtered, and slowly evaporated and crystallized at 22 °C. Colorless block crystals were obtained after 12 days.

For the preparation of MAT-26DHB (1:1), 0.2 mmol MAT (49.6 mg) and 0.2 mmol 26DHB (30.8 mg) were weighed into a mortar and 1 mL of ethyl acetate was added for grinding to obtain a powder sample. The prepared powder sample was dissolved in acetone, filtered, and slowly evaporated and crystallized at 22 °C. Colorless block crystals were obtained after 10 days.

For the preparation of MAT-SAL-H_2_O (1:1:1), 0.2 mmol MAT (49.6 mg) and 0.2 mmol SAL (27.6 mg) were weighed into a mortar and 0.5 mL of ethyl acetate was added for grinding to obtain a powder sample. The prepared powder sample was dissolved in acetone, filtered, and slowly evaporated and crystallized at 22 °C. Colorless block crystals were obtained after 10 days.

#### 3.2.2. SXRD Analysis

The SXRD data were collected on a Rigaku XtaLAB-DW apparatus (Rigaku, Tokyo, Japan) with Cu–Kα radiation (λ = 1.54178 Å) at 295 K. Crystal structures were solved and refined through direct methods by using Olex 2 (1.5) and SHELXL software (2018.3) [41,42]. All H atoms were refined isotropically and placed in calculated positions through geometric calculation.

#### 3.2.3. PXRD Analysis

The PXRD experiments were performed on a Rigaku SmartLab apparatus (Rigaku, Tokyo, Japan) with Cu Kα radiation (λ = 1.54178 Å). The equipment continuously scanned samples over the 2θ range of 3°–80° with a step size of 0.02° at a constant scan rate of 8°/min. Data were processed by using JADE 6.0 software. Mercury software (2023.1.0) [43] was used to calculate the PXRD simulations.

#### 3.2.4. Thermal Analysis

The DSC curves of all samples were collected with a Mettler Toledo DSC instrument (Greifensee, Switzerland). A total of 3–5 mg of a sample was weighed, placed in a standard aluminum pan, heated at 10 °C·min^−1^, and scanned over the range of 30–300 °C under ambient air.

The TG curves of all samples were collected by using a Mettler Toledo DSC instrument (Greifensee, Switzerland). A total of 5–8 mg of a sample was weighed, placed in an alumina crucible, and heated at 5 °C·min^−1^. Nitrogen was used as the protective gas with a gas flow rate of 50 mL·min^−1^. The samples were scanned at 30–500 °C.

All spectra were analyzed and processed by STARE (Evaluation software 16.30) software.

#### 3.2.5. IR Analysis

Spectral analysis was performed with a Spectrum 400 Fourier transform infrared spectrometer (PerkinElmer, Waltham, MA, USA) with a spectral scanning range of 400–4000 cm^−1^ at a resolution of 4 cm^−1^ for 16 times.

#### 3.2.6. Solubility Tests

The equilibrium solubility of MAT and its three salts in purified water, phosphate buffer (pH 6.8, configured by 0.050 mol·L^−1^ KH_2_PO_4_ and 0.024 mol·L^−1^ NaOH), acetic acid buffer (pH 4.5, configured by 0.22 mol·L^−1^ CH_3_COONa and 0.17 mol·L^−1^ CH_3_COOH), and 0.10 mol·L^−1^ hydrochloric acid solution (pH 1.2) was determined. Excess samples were placed in 5 mL glass vessels containing 1 mL of dissolution media in parallel for three parts, stirred at 37 °C at 160 rpm for 72 h, and then left for 1 day at an ambient humidity of 68%. The concentration of the samples was analyzed through high-performance liquid chromatography. The mobile phase consisted of methanol, acetonitrile, and phosphate buffer (15:15:70). Detection was performed at 220 nm at the flow rate of 1.0 mL/min and the injection volume of 10 μL [44].

#### 3.2.7. Theoretical Computation

A Gaussian package [45] was used for the geometry optimizations and single-point energy calculation. The theoretical levels were B3LYP-D3BJ/6-31G (d, p) and M06-2X/def2-TZVP. Only the coordinates of the hydrogen atoms were optimized and those of the heavy atoms were maintained on the basis of the SXRD results. The solvent-free energy was calculated at the level of M06-2X/6-31G* by using the solvation model based on density (SMD) in accordance with references [46,47]. The lattice energy was calculated by utilizing CRYSTAL 17 software [48]. A Hirshfeld surface analysis was conducted with Crystal Explore 21.5 software [49].

## 4. Conclusions

In this work, the three novel salt forms MAT-25DHB, MAT-26DHB, and MAT-SAL-H_2_O were prepared. They were characterized by using SCXRD, PXRD, DSC, TG, and IR technologies. The crystal packing similarity analysis revealed that the conformations of the main molecule of MAT and the guest molecule (benzoic acid part) were basically the same. The results of the stability evaluation illustrated that the melting points of the three kinds of salts improved. Analyzing the melting points of the previously reported MAT salts revealed that no obvious correlation existed between the melting point of the obtained salt and that of the SF. The melting points of MAT-25DHB reported in this paper was higher than those of the SFs and MAT, and it is the most stable salt reported at present. Compared with the aqueous solubility of MAT, the solubility of MAT-25DHB was significantly higher, whereas that of MAT-26DHB and MAT-SAL-H_2_O was lower. Subsequently, theoretical calculations were conducted to explain the reasons for the changes in stability and solubility from the perspective of energy. The results show that although the structures of 25DHB and 26DHB are very similar, there are large differences in the free energy of solvents, which is presumed to be the main reason for the different solubility of the corresponding salts. From the overall results of this study, MAT-25DHB salt is better than the other salt forms in terms of thermal stability and aqueous solubility. Therefore, MAT-25DHB is expected to undergo further research as an optimized salt. In addition, the new structures reported herein could provide certain reference values for the subsequent development and utilization of MAT.

## Figures and Tables

**Table 1:** Crystallographic data and structural refinement parameters of MAT-25DHB, MAT-26DHB, and MAT-SAL-H_2_O.

Parameters	MAT-25DHB	MAT-26DHB	MAT-SAL-H_2_O
Empirical formula	(C_15_H_25_N_2_O)^+^·(C_7_H_5_O_4_)^−^	(C_15_H_25_N_2_O)^+^·(C_7_H_5_O_4_)^−^	(C_15_H_25_N_2_O)^+^·(C_7_H_5_O_3_)^−^·H_2_O
Formula weight (Da)	402.49	402.49	386.49
Crystal size(mm)	0.30 × 0.40 × 0.40	0.20 × 0.20 × 0.20	0.20 × 0.20 × 0.20
Description	block	block	block
Crystal system	orthorhombic	orthorhombic	orthorhombic
Space group	*P* 2_1_2_1_2_1_	*P* 2_1_2_1_2_1_	*P* 2_1_2_1_2_1_
a (Å)	9.5462(2)	8.8803(2)	9.3422(19)
b (Å)	13.9464(3)	13.4444(2)	12.7748(2)
c (Å)	15.1799(3)	17.3365(3)	17.7168(3)
α (°)	90	90	90
β (°)	90	90	90
γ (°)	90	90	90
Z	4	4	4
Volume (Å^3^)	2020.98(8)	2069.81(8)	2114.41(7)
Density(g/cm^3^)	1.323	1.292	1.271
R_1_	0.0484	0.0361	0.0524
wR_2_	0.1259	0.0958	0.1540
Reflections collected	4045	3894	4016
independent reflections	3835	3442	3582
Completeness (%)	99.7	100	99.9
CCDC number	2,297,779	2,297,780	2,297,778

**Table 2:** Parameters of the main hydrogen bonds of the salts.

Salt	D–H…A	d(D–H)/Å	d(H…A)/Å	d(D…A)/Å	∠DHA/°
MAT-25DHB	O_2B_–H_2B_…O_3B_	0.820	1.793	2.517(4)	146.34
	N_2A_–H_2A_…O_4B_	0.980	1.756	2.719(3)	166.51
	O_1B_–H_1B_…O_1A_	0.820	1.899	2.653(4)	152.39
MAT-26DHB	O_1B_–H_1B_…O_2B_	0.820	1.809	2.541(3)	147.92
	O_4B_–H_4B_…O_3B_	0.820	1.801	2.536(3)	148.50
	N_2A_–H_2A_…O_2B_	0.980	1.802	2.764(3)	166.53
MAT-SAL-H_2_O	O_3B_–H_3B_…O_2B_	0.820	1.784	2.517(4)	148.00
	O_1W_–H_1W_…O_1A_	0.877	1.982	2.855(4)	173.14
	O_1W_–H_1W_…O_1B_	0.855	2.058	2.907(4)	172.50
	N_2A_–H_2A_…O_1B_	0.980	1.799	2.761(4)	166.53

**Table 3:** Equilibrium solubility of MAT and its salts (*n* = 3).

Samples	Concentration (mg/mL)			
	H_2_O	pH = 1.2	pH = 4.5	pH = 6.8
MAT	65.36 ± 0.53	90.89 ± 1.01	91.79 ± 2.31	64.89 ± 1.13
MAT-SAL-H_2_O	29.00 ± 1.28	55.89 ± 6.54	40.07 ± 1.15	25.09 ± 0.82
MAT-26DHB	7.42 ± 0.45	13.08 ± 1.52	8.23 ± 0.10	5.71 ± 0.75
MAT-25DHB	613.60 ± 62.57	795.11 ± 18.28	606.03 ± 36.97	482.55 ± 31.93

**Table 4:** Lattice energies of three salts.

Salts	E (Bulk)	E (Mol, Bulk)	E (Mol, Ghost)	BSSE	E (Lattice)
MAT-SAL-H_2_O	−5368.4772	−1342.0654	−1342.0955	0.0301	−14.90
MAT–26–DHB	−5363.7148	−1340.8063	−1340.8063	0.0000	−76.80
MAT–25–DHB	−5363.6795	−1340.7405	−1340.7794	0.0389	−88.13

**Table 5:** Symbol and the corresponding meaning.

	Symbol	Physical Meaning
1	ΔE (Lattice)	the lattice energy
2	∆E (Condensation)	the condensation of molecules that retain the same conformation they had in the crystal
3	BSSE	the basis set superposition error
4	E (Bulk)	the total energy per unit cell
5	Z	the number of molecules in the cell
6	E (Molecule, Bulk)	the total energy of each molecule in the cell
7	E (Molecule, Ghost)	the total energy of the molecule obtained by increasing the basis group with the virtual function of the surrounding atoms
8	ΔG (solv)	Solvation free energy
9	E (soln)	the single-point energy in the solvent
10	E (gas)	the single–point energy in the gaseous state

## Data Availability

CCDC 2297778, 2297779 and 2297780 contain the supplementary crystallographic data for this paper. These data can be obtained free of charge via www.ccdc.cam.ac.uk/data_request/cif, or by emailing data_request@ccdc.cam.ac.uk, or by contacting The Cambridge Crystallographic Data Centre, 12 Union Road, Cambridge CB2 1EZ, UK; fax: +441223 336033.
